# Reference Paper: Maroun et al. “Endoscopic discectomy for L4-L5 disc herniation: A meta-analysis comparing transforaminal and interlaminar approaches”

**DOI:** 10.1016/j.bas.2026.106023

**Published:** 2026-03-25

**Authors:** Suthipas Pongmanee, Jin-Sung Kim

**Affiliations:** Department of Orthopedics, Faculty of Medicine, Chiang Mai University, Chiang Mai, Thailand; Department of Neurosurgery, Seoul St Mary's Hospital, College of Medicine, The Catholic University of Korea, Seoul, South Korea

Dear Editor,

We read with great interest the meta-analysis by Maroun et al. comparing transforaminal (TF) and interlaminar (IL) approaches for L4-L5 disc herniation. The authors have provided a valuable level-specific analysis addressing a critical gap in the endoscopic spine surgery literature. However, we propose a reinterpretation of their findings through the lens of established anatomical algorithms that fundamentally changes the clinical implications of their work.

The Maroun analysis includes six studies comprising 456 patients total (276 TF, 180 IL). While the authors concluded that "both approaches produce similar results regarding overall complications, reoperation rates, operative time, LOS, and patient-reported outcome measures," the extreme heterogeneity reported in the operative time analysis (I^2^ = 95%) warrants reexamination. Rather than treating heterogeneity as a nuisance parameter to be accommodated through random-effects modeling, we argue that this heterogeneity represents a signal—not noise—indicating distinct underlying clinical scenarios.

Consider the Huang et al. (2021) study, included in the Maroun analysis, which specifically examined highly down-migrated L4-L5 disc herniations. The results reveal a striking divergence from other included studies: the IL approach required only 41.8 minutes compared to 50.3 minutes for the TF approach (p = 0.045), with significantly shorter fluoroscopy time (1.8 min vs. 13.7 min). In contrast, studies likely including simpler paracentral herniations without superior migration reported faster TF times with minimal laminotomy requirements. When these contradictory findings are pooled without stratification, the reported mean difference of 0.73 minutes (95% CI −14.83 to 16.29, p = 0.93) becomes an artifact of averaging opposing vectors.

This heterogeneity becomes explicable and clinically coherent when reframed through the Kotheeranurak et al. (2023) systematic review and consensus algorithm, published in *Spine* with international expert input. The algorithm proposes a structured framework for approach selection based on zonal anatomy: TF is indicated for foraminal and extraforaminal pathology, while IL is preferred for central, subarticular, or migrated discs. At L4-L5, where both foraminal and central pathologies occur, both approaches have distinct and appropriate roles—not because they are equivalent in all scenarios, but because they are specialized for distinct anatomical presentations.

Furthermore, comparison with broader multi-level meta-analyses provides essential context that the Maroun analysis does not fully engage with the anatomical implications of these data. Jitpakdee et al. (2023), published in the *Global Spine Journal*, analyzed 18 studies involving 1948 patients across multiple lumbar levels. Critically, this larger analysis identified that the TF approach required significantly more operative time and radiation exposure at the L5-S1 level compared to the IL approach—a finding attributed to the high iliac crest complicating transforaminal access at that level. This validates the anatomical hypothesis: approach performance is not independent of level-specific anatomy.

The Maroun authors, by restricting their analysis to L4-L5, appropriately removed the confounding effect of L5-S1 anatomy. However, they did not incorporate stratification by disc migration grade or anesthetic protocol to account for the residual heterogeneity at L4-L5. While Jitpakdee et al. (2023) is cited in the Maroun discussion, it is referenced primarily to corroborate the equivalence findings rather than to engage with its broader implications regarding anatomy-specific approach performance. More critically, the Kotheeranurak et al. (2023) consensus algorithm—directly relevant to approach selection at L4-L5—is absent from the discussion, depriving readers of the contextual framework in which simple equivalence is known to break down under specific anatomical constraints.

We propose that the Maroun meta-analysis would be substantially strengthened by reframing its primary conclusion. Rather than "both approaches produce similar results," a more accurate and clinically useful interpretation would be: "When appropriately selected based on pathology location and patient anatomy, both the TF and IL approaches achieve comparable functional outcomes, supporting the use of established anatomical algorithms for approach selection." This subtle but important distinction transforms the analysis from seeming to recommend interchangeability to validating anatomically-guided, algorithm-based decision-making.

For future meta-analytic efforts in endoscopic spine surgery, we recommend pre-planned subgroup analyses stratified by: (1) disc location (foraminal vs. paracentral vs. central vs. migrated, using established zonal classification systems), (2) migration grade (using recognized migration scales, e.g., zones 1-4), (3) surgeon experience level and case volume, and (4) institutional anesthetic protocols (local vs. general anesthesia). Such stratification would transform comparative meta-analyses from binary "equivalence" conclusions to nuanced, anatomy-guided frameworks that meaningfully advance clinical decision-making.

We commend the Maroun team for their contribution to endoscopic spine surgery literature and recognize that limitations in the primary study data constrain what meta-analyses can definitively conclude. We believe this alternative interpretation, grounded in established consensus algorithms and validated by larger comparative analyses, provides a more clinically useful and scientifically rigorous framework for interpreting their findings.

## Declaration of competing interest

The authors declare the following financial interests/personal relationships which may be considered as potential competing interests: Jin-sung Kim reports was provided by The Catholic University of Korea Seoul St Mary's Hospital. Suthipas Pongmanee reports was provided by Chiang Mai University Faculty of Medicine. If there are other authors, they declare that they have no known competing financial interests or personal relationships that could have appeared to influence the work reported in this paper.
